# Ascorbate and Thiamin: Metabolic Modulators in Plant Acclimation Responses

**DOI:** 10.3390/plants9010101

**Published:** 2020-01-13

**Authors:** Laise Rosado-Souza, Alisdair R. Fernie, Fayezeh Aarabi

**Affiliations:** Max-Planck-Institut für Molekulare Pflanzenphysiologie, Am Mühlenberg 1, 14476 Potsdam-Golm, Germany; Rosado@mpimp-golm.mpg.de

**Keywords:** metabolite signaling/acclimation, TCA cycle, Calvin–Benson cycle, photoperiodic changes, photosynthesis, redox-regulation, environmental adaptation

## Abstract

Cell compartmentalization allows incompatible chemical reactions and localised responses to occur simultaneously, however, it also requires a complex system of communication between compartments in order to maintain the functionality of vital processes. It is clear that multiple such signals must exist, yet little is known about the identity of the key players orchestrating these interactions or about the role in the coordination of other processes. Mitochondria and chloroplasts have a considerable number of metabolites in common and are interdependent at multiple levels. Therefore, metabolites represent strong candidates as communicators between these organelles. In this context, vitamins and similar small molecules emerge as possible linkers to mediate metabolic crosstalk between compartments. This review focuses on two vitamins as potential metabolic signals within the plant cell, vitamin C (L-ascorbate) and vitamin B_1_ (thiamin). These two vitamins demonstrate the importance of metabolites in shaping cellular processes working as metabolic signals during acclimation processes. Inferences based on the combined studies of environment, genotype, and metabolite, in order to unravel signaling functions, are also highlighted.

## 1. Introduction

The different cellular compartments such as chloroplasts, mitochondria, and the nucleus require a tightly orchestrated coordination of metabolic activity within each compartment by anterograde and retrograde signaling pathways [1,2]. Anterograde signaling is essentially a top-down regulatory pathway originated in the nucleus and sent to the organelles. Retrograde signaling, on the other hand, is the ability of organelles to coordinate, via signaling molecules, the expression of nuclear genes [3,4].

During the past years, significant advances in uncovering specific signals from plastids and their mechanisms of action have been made [4,5]. Several classes of factors including organelle gene expression, redox status, accumulation of pigment precursors like tetrapyrroles, reactive oxygen species (ROS), and metabolites have been proposed to act as plastidial signals [1]. They are sensed by plastidial factors including Executer1, Executer2, Genomes Uncoupled 1 (GUN1), and a thylakoid protein kinase (STN7), to initiate signaling cascades [1,4]. Some nuclear factors such as the transcription factor abscisic acid insensitive 4 (ABI4) have additionally been identified to be involved in retrograde signaling [6].

Much less is known about plant mitochondrial retrograde regulation/signaling [7,8]. Current research focuses on the response to a dysfunctional mitochondrial electron transport chain (ETR) and activation of genes encoding enzymes associated with the recovery of mitochondrial function, such as alternative oxidase (AOX) and alternative NAD(P)H dehydrogenases. It is also known that genes encoding proteins associated with the maintenance of the redox homeostasis, such as glutathione reductase, catalases, ascorbate peroxidases, and superoxide dismutases, are affected [9]. Recently an additional key player in the coordination between chloroplast and mitochondrial signaling pathways has been identified by Shapiguzov and coworkers (2019); their results suggest that the nuclear protein radical-induced cell death1 (RCD1) combines the signaling from both organelles in order to govern transcriptional and metabolic process within each organelle [10,11]. RCD1 mediates this regulation by suppressing the abscisic-acid-responsive NAC (ANAC) transcription factors ANAC013 and ANAC017, known as regulators of the mitochondrial dysfunction stimulon (MDS) genes, and also by receiving the ROS signals from the chloroplast underging protein modifications [10,11].

Many studies have emphasized the high degree of interrelationship between photosynthesis and respiration, the major energy production pathways that are confined to the chloroplast and mitochondria, respectively [12,13,14]. Metabolite signals are now frequently proposed as potential signals for inter-organellar communication and possible modulators to support photosynthesis during acclimation to fluctuating environments [3,15,16].

The focus of this review is on two vitamins, vitamin C (L-ascorbate) and vitamin B_1_ (thiamin), as potential metabolic signals within the plant cell, and to summarize recent advances on their roles in plant acclimation responses.

Vitamins in general are essential for plant metabolism, because many of them display important redox chemistry and antioxidant potential or are used as cofactors in several enzymatic reactions. Cartenoids (Pro-vitamin A), ascorbate, vitamin E (both tocopherols and tocotrienols), and vitamin B compounds (such as thiamin) are known to have predominant antioxidant roles in plants under oxidative stresses. Plastids are organelles highly exposed to oxidative stress because of oxygenic photosynthesis, and thus are protected by antioxidant vitamins, as reviewed in Asensi-Fabado and Munne-Bosch, 2010 [17].

Ascorbate is known as the most abundant and ubiquitous cellular antioxidant and is present in most cellular compartments [18]. The antioxidant function of ascorbate is mainly attributed to its action as a substrate for the ascorbate–glutathione cycle in scavenging hydrogen peroxide [19]. Ascorbate is also used as a cofactor for the violaxanthin de-epoxidase (VDE) enzyme, a critical component of the non-photochemical quenching (NPQ) [18]. Having the profound antioxidant functions to scavenge ROS renders ascorbate an important metabolite in the plant acclimation responses to changing environments [20,21]. For instance, ascorbate has been demonstrated to accumulate in Arabidopsis leaves during the acclimation process following the transition from low to high light conditions [21], as well as in the leaves of highland species and pea acclimated to high light and low temperature [22]. Ascorbate is connected to the mitochondria and the respiration processes, because the last enzyme of the pathway is located in the inner membrane of the mitochondria; however, it is found to be almost ubiquitously scattered in all cellular compartments, including chloroplast [23]. Further, ascorbate is known as a key component of the redox hub in balancing redox homeostasis in cellular compartments [19], and owing to the fact that redox equivalents can also be transferred between cellular compartments, ascorbate is, therefore, assumed as part of the inter-organellar communication.

Thiamin (or thiamine), also known as vitamin B_1_, is one of the water-soluble B-complex vitamins. The term refers to the three vitamers forms, free thiamin; thiamin monophosphate (TMP); and thiamin pyrophosphate (TPP, or thiamin diphosphate, TDP), which is the active form. TPP works as an essential coenzyme for enzymes involved in photosynthesis in chloroplasts, in ATP synthesis in the participation in oxidative decarboxylation of pyruvate, and in the tricarboxylic acid cycle in mitochondrial central metabolism, as well as in the pentose phosphate pathway and alcoholic fermentation in cytoplasm [24,25,26,27]. Thiamin has also been shown to be involved in the acclimation responses to abiotic stresses and photoperiod [28,29,30,31,32]. It plays important roles, working directly as an antioxidant, scavenging ROS, and protection molecule, and indirectly by contributing to the cell energy poll, conferring the cell the necessary metabolic flexibility to acclimate to new conditions [17,32,33].

In the next sections, a detailed description of each pathway and their roles in plant acclimation responses to environmental cues, in particular high light and photoperiod acclimation, are discussed.

## 2. Ascorbate Biosynthesis and Subcellular Distribution

Ascorbate is present in Arabidopsis leaves as one of the most abundant primary metabolites [18]. In plants, ascorbate is generally synthesized through one dominant pathway, so-called the D-mannose/L-galactose (Smirnoff–Wheeler) pathway [18]. Three other pathways, so-called the *Myo*-inositol [34], L-gulose [35], and L-galacturonate [36], have been also suggested as alternative routes for ascorbate biosynthesis [37]. However, strong evidence on the existence of all these alternative pathways has not yet been reported. Moreover, it appears that even if the alternative pathways exist, their roles in ascorbate biosynthesis should be minor at least in Arabidopsis, where ascorbate loss in the *vtc2vtc5* double mutants appears not to be compensated by the other pathways [38].

**Figure 1:**
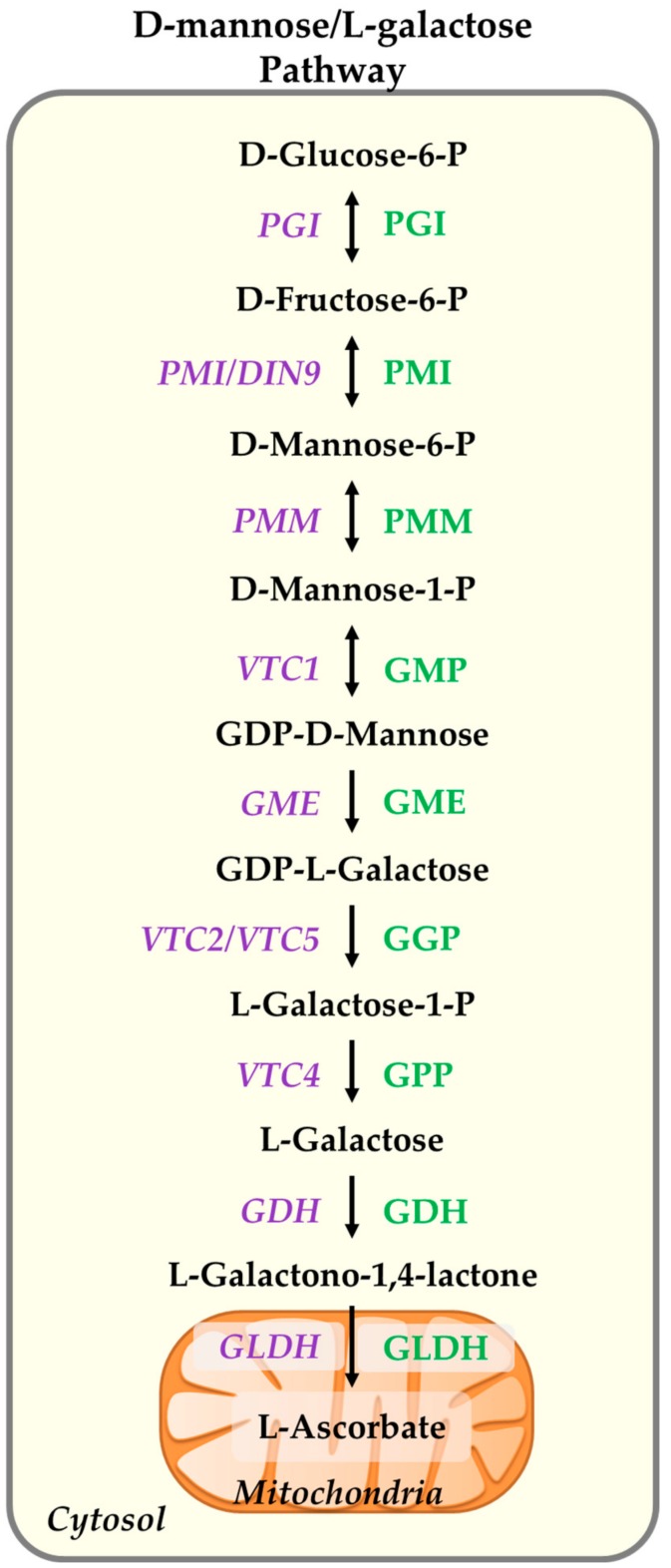
D-Mannose/L-Galactose pathway of ascorbate biosynthesis in plants. The genes of the pathway are highlighted in purple and written in italics. The enzymes are highlighted in green. Phosphoglucose isomerase (PGI), phosphomannose isomerase (PMI), and phosphomannomutase (PMM) are responsible for the conversion of D-glucose-6-P to D-mannose-1-P, the direct precursor of GDP-D-mannose pyrophosphorylase (GMP), the first committed enzyme of the pathway encoded by *VTC1*. GDP-mannose-3′-5′-epimerase (GME), GDP-L-galactose transferase (GGP), L-galactose-1-phosphate phosphatase (GPP), L-galactose dehydrogenase (GDH), and L-galactono-1,4-lactone dehydrogenase (GLDH) are the next enzymes of the pathway. GGP is the key enzyme of the pathway encoded by *VTC2* and *VTC5* paralogs. This enzyme undergoes feedback regulation by ascorbate pool size. GLDH is located in the intermembrane of mitochondria and is connected to the mitochondria respiratory chain.

Therefore, here, we only demonstrate the details of the major pathway (Figure 1). In this pathway, D-glucose-6-phosphate is converted to ascorbate in nine enzymatic reactions, as depicted in Figure 1, with the last step catalized by L-galactono-1,4-lactone dehydrogenase (GLDH), located in the inner membrane of the mitochondria in which L-galactono-1, 4-lactone, the direct precursor of ascorbate, is converted to ascorbate.

Ascorbate specific immunogold labelling and quantitative transmission electron microscopy showed that ascorbate was found in most cellular organelles, including cytosol, nuclei, peroxisomes, vacuoles, mitochondria, and chloroplasts, but not in cell walls and intercellular spaces. Moreover, it has been shown that, despite showing a strong increase in chloroplasts (104%) under high light conditions (700 μmol m^-2^s^−1^), vacuoles even demonstrated a stronger ascorbate specific labeling (395%) than chloroplasts. This highlights the relevance of vacuoles in ascorbate metabolism in response to high light acclimation, which deserves further investigations [23].

Given that ascorbate distributes across all the cellular compartments, despite exclusive production in mitochondria [39], the involvement of ascorbate transporters is necessary for its function. The identification of ascorbate transporters has long been considered as a difficult task [40] but eventually, a phosphate transporter 4 family protein (*At*PHT4;4) was identified as an ascorbate transporter located at the chloroplast envelope membrane [41]. However, transporters localized to other membranes remain unknown and information concerning subcellular ascorbate concentration is rare and normally confined to single environmental conditions.

## 3. Role of Ascorbate in Light Acclimation

Under the light acclimation process, the chloroplast undergoes coordinated metabolic adjustments with extra-chloroplastic metabolism in order to maintain the overall fitness of plants and avoid damage [42]. Several metabolites produced in the plastids and motochondria are subsequently transmitted to the nucleus and modulate nuclear gene expression. This phenomenon is termed as retrograde signaling and known as a critical component of plant acclimation responses. Several light-shift experiments have been conducted to unravel the early and late metabolic responses to different light intensities. Changes in light intensity rapidly manipulate the electron pressure generated in the photosynthetic electron transport chain (pETC); therefore, the ROS signals generated from pETC are considered as important retrograde signals for short- and long-term acclimation [42]. Ascorbate and glutathione are known as redox signals, playing roles on longer time scales [42]. It has been revealed that total ascorbate levels increased after an hour in plants exposed to high light (800 μmol photons m^−2^ s^−1^). This increase is even delayed by 3 h in plants transferred to high light following acclimation to low light intensity (8 μmol photons m^−2^ s^−1^) [43].

One important function of ascorbate is as a cofactor in the xanthophyll cycle, in which the excess excitation energy is dissipated as heat from excited chlorophylls to xanthophyll carotenoids, a photoprotection mechanism termed as non-photochemical fluorescence quenching (NPQ) [44]. In this cycle, the violaxanthin de-epoxidase (VDE) enzyme, localized in the thylakoid lumen, uses ascorbate as a cofactor to reduce the epoxide group of the substrate violaxanthin and converts it to antheraxanthin and zeaxanthin [45]. In a light-shift experiment (from 160 μmol photons m^−2^ s^−1^ to 1800 μmol photons m^−2^ s^−1^), roles of ascorbate in light acclimation were investigated using the Arabidopsis mutant deficient in VDE enzyme (*npq1)*, ascorbate deficient mutant (*vtc2)*, along with *vtc2npq1* double knockouts [46]. It has been revealed that the *vtc2* mutants, having 10%–30% of the wild type (WT) ascorbate levels, lost their acclimation capacity after long-term exposure to high light (up to five days at 1800 μmol photons m^−2^ s^−1^). In contrast to the *npq1* single mutants, deficient in zeaxanthin, which were slightly more sensitive to high light than the WTs, *vtc2* and *vtc2npq1* double mutants showed an increased degree of bleached leaves, lipid peroxidation, and photoinhibition (increased degree of damage to (Photosystem II) PSII, measured by Fv/Fm). These data confirmed the importance of ascorbate in light acclimation responses and also showed that ascorbate has even more important roles than other photoprotective metabolites such as xanthophylls in acclimation to high light stress. Further, loss of PSII efficiency was not observed after short-term high light exposure (up to 2 h) in *vtc2* mutants, however, the conversion rate of violaxanthin to zeaxanthin was reduced owing to the dependency of VDE to ascorbate [47]. These data further corroborated the importance of ascorbate on long-term acclimation to high light rather than short-term.

In a subsequent study in which they investigated the thylakoid-associated proteome of Arabidopsis WT and *vtc2* after transition to high light (1000 μmol photons m^−2^ s^−1^), differential protein accumulation could be observed in a number of stress-associated proteins between WT and *vtc2* including Fe-superoxide dismutase (Fe-SOD), Cu, Zn-SOD, HSP70s (cpHSP70-1 and 2), PsbS protein, and a chloroplast-localized glyoxalate I [48]. SODs are metalloenzymes, which have been long known as stable markers for abiotic stress tolerance against ROS [49]. Also, it has been shown that HSP70-2 in *Chlamydomonas reinhardtii* chloroplasts has photoprotective roles for PSII reaction centers during photoinhibition and PSII repair [48,50]. Apart from the xanthophyll zeaxanthin, PsbS is known as another component of NPQ [51]. PsbS-dependent quenching site has been recently deciphered to be in Light-harvesting complex II (LHCII), and in the PSII core, most likely in the core antenna complexes CP43 and/or CP47 [52]. In the study of Giacomelli and coworkers, PsbS protein was up-regulated more than twofold upon transition to high light, however, it remained unchanged in the *vtc2*, which is in line with the observation that *vtc2* mutants have reduced levels of non-photochemical quenching [47]. This study shows that ascorbate has a significant impact on chloroplast proteome linking to oxidative stress and quenching, however, it cannot be entirely ruled out that these changes are the consequences of a direct or indirect effect of ascorbate deficiencies in the *vtc2* mutants. Moreover, the ascorbate deficient mutants, *vtc1*, *vtc2*, and *vtc3*, were found to accumulate visibly and quantitatively less anthocyanin compared with the wild types during the high light treatment in several studies [48,53,54]. *vtc1* and *vtc2* mutants were also unable to induce the expression of anthocyanin biosynthesis enzymes, and the corresponding transcription factors of the pathway, PAP1, GL3, and EGL3 under high light acclimation [54], whereas the transcripts related to anthocyanin biosynthesis and regulation are known to be up-regulated rapidly by high light in Arabidopsis WT plants [54,55]. Further, given the fact that both ascorbate and anthocyanin have been shown to accumulate in a similar time-scale (days) and in similar ranges of light intensities after high light exposure, and that the *vtc* mutants had defects in the accumulation of anthocyanin, the existing interconnection between them has been proposed in the study of Page and coworkers [54]. The authors observed a tight correlation of ascorbate and anthocyanin levels across six different Arabidopsis ecotypes under normal and high light conditions, which adds further proof to the relationship between them [54]. More investigation should be done to explore the co-regulatory mechanism of ascorbate levels with anthocyanin under high light acclimation.

## 4. Light Regulation of Ascorbate

Despite existing cumulative evidence on the importance of ascorbate on light acclimation responses, regulatory mechanisms of the ascorbate pool size by light remained poorly understood. It appears that ascorbate pool size is highly sensitive to both the light intensity and time of the day because transcript profiles of the genes encoding the enzymes of the pathway behaved unpredictably in different light-shift experiments and vary between species. Therefore, conclusions on the correlation between the gene expression, activity of the corresponding enzymes of the pathway, and the ascorbate pool size are inconsistent between studies. That being said, the comparison of multiple light-shift experiments revealed GDP-L-galactose phosphorylase (GGP) as the key enzyme of the pathway controlling the ascorbate levels under high light [54,56,57,58,59]. The corresponding genes encoding this enzyme, the first committed step of the ascorbate biosynthetic pathway, are *VTC2* and *VTC5* paralogs, which were identified to be induced in concert upon 24 h exposure to high light, leading to a 20-fold increase in the activity of the corresponding enzyme, and an increase in ascorbate levels [56].

In a study where the authors explored the transcriptional regulation of ascorbate by RNA-seq following a step change of light intensity in Arabidopsis, *VTC2* and, to lesser extent, *VTC5* were validated as regulatory points in light accumulation of ascorbate, the expression of both genes were correlated with different light intensities, however, a minor change in GDP mannose pyrophosphorylase (GMP) could also be observed [57]. Moreover, GGP has been proposed as a key rate-limiting step for ascorbate biosynthesis not only in Arabidopsis [60], but also in other species including tobacco [61], apple [62], and kiwifruit [60,63]. Besides, the light-responsiveness of *VTC2* expression has been observed in tomato fruits following an observation on *VTC2* reduction under a continuous shading [64]. GGP has also been validated as a highly regulated enzyme in the green algea, *Chlamydomonas reinhardtii,* where it is thought to exhibit protective function against oxidative stress [65,66]. Further, a potential regulatory role for GGP has been proposed owing to the evidence on nuclear localization of the protein; however, as yet, no mechanistic evidence proposed has been supposed as a hypothesis [58].

Besides GGP, GLDH has been also suggested as an important controlling point for light regulation of ascorbate biosynthesis at the level of the enzyme activity [67,68]. Arabidopsis plants, grown under high light after supplementation with L-galactone-1,4-lactone (L-Gal; the precursor of ascorbate), accumulated up to twofold ascorbate levels and had twice as high GLDH activities of the low-light grown plants, assumed as higher respiration rates [69]. GLDH is located in the inner membrane of the mitochondria, which carries a redox-sensitive thiol residue (Cys-340), critical for the conversion of L-Gal into ascorbate [70]. This residue has been validated to be irreversibly oxidized by H_2_O_2_, inactivating GLDH [70], and has been suggested to be responsible for regulation of GLDH activity during the early stages of heat stress produced programmed cell death [67,71]. Moreover, Arabidopsis *GLDH* overexpressing lines accumulated higher ascorbate levels and demonstrated higher chlorophyll fluorescence parameters after exposure to high light for 14 days, which led them to have lower sensitivity to light stress [72].

It should be noted that, despite observing multiple studies on light effects on the ascorbate biosynthetic pathway, so far, few reports exist concerning the effects of light on the components of ascorbate recycling and turnover [73]. One report, however, does demonstrate that the activities of dehydroascorbate reductase and monodehydroascorbate reductase are enhanced in the Arabidopsis plants, grown under high light [69].

### 4.1. Transcriptional Regulation

Despite observing alterations in gene expression patterns of the ascorbate biosynthetic pathway under light stress in multiple studies, the upstream signal transduction pathway controlling this phenomenon is largely unknown. Studies were performed to decipher light-regulated *cis-*elements in rice [74] and subsequently in Arabidopsis [75]. The conserved sequences (the GT1 box and the TGACG motif) in the promoter regions of the *L-galactose-1-phosphate phosphatase (GPP*) and *GLDH* genes were found to be responsible for light induction of these genes in rice [74]. Further efforts have been made to find such consensus elements in Arabidopsis, however, authors identified a different, but critical region for light regulation of *VTC2*, in –40 to –70 bp of its promoter [75]. Information on whether such a casual promoter region exist upstream of other genes of the pathway is not yet available.

Ascorbic acid mannose pathway regulator 1 (AMR1) has been identified as a negative regulator of multiple genes encoding early and late enzymes of the Man/L-Gal pathway, including GMP, GME, GGP, GPP, GDH, and GLDH, with the highest effect on GME and GGP [76]. The expression of AMR1 has been validated to be decreased by light and to be accompanied by an increase in ascorbate levels [76]. In contrast to AMR1, the ethylene response factor98 (*At*ERF98) has been identified as a positive regulator of D-Man/L-Gal pathway, by directly binding to the promoter of *VTC1*; encoding GMP; and also enhancing the expression of multiple genes of the pathway including, *VTC1*, *VTC2*, *GDH,* and *GLDH* [77]. Although the essential role of ERF98 has been revealed under the salt stress [77], no investigations have been done to verify its role under the light stress.

**Figure 2:**
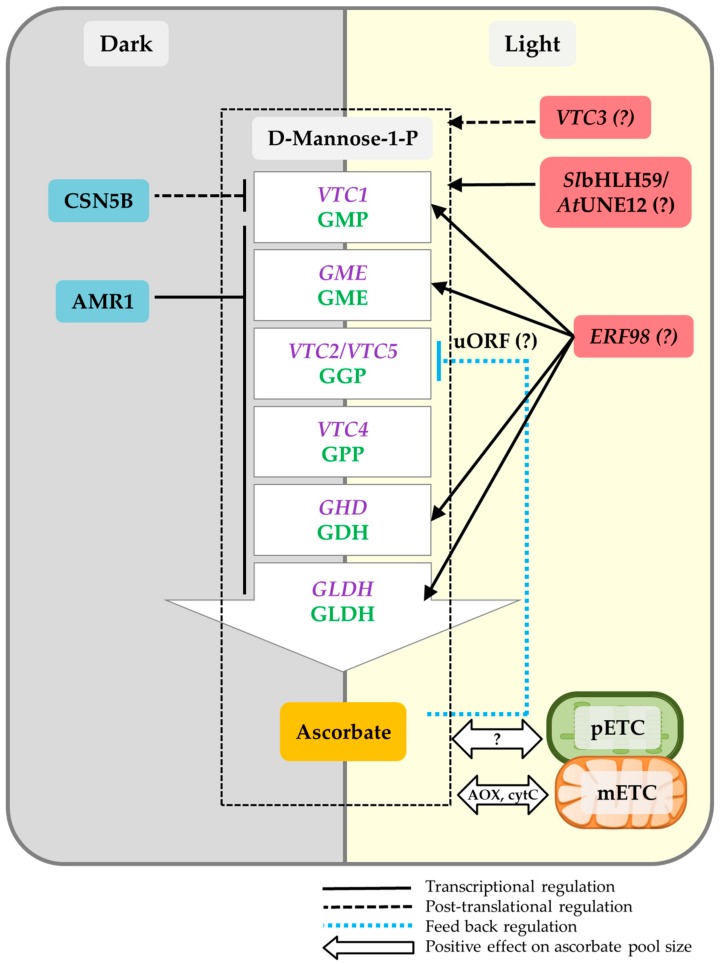
Overview of light regulation on ascorbate biosynthesis. Ascorbate biosynthesis is regulated transcriptionally by the ascorbic acid mannose pathway regulator 1 (AMR1), ethylene response factor 98 (ERF98), and *Solanum lycopersicum* basic helix–loop–helix (bHLH) transcription factor 59 (*sl*bHLH59; tomato-specific). AMR1 has negative effects on *GMP*, *GME*, *GGP*, *GPP*, *GDH*, and *GLDH* in Arabidopsis. *AMR1* expression decreases rapidly under light; thereafter, ascorbate levels increase. *At*ERF98 is the positive regulator of the ascorbate pathway by directly binding to the promoter of *VTC1*, and up-regulating the expression of *VTC1*, *VTC2, GDH*, and *GLDH.* The light-specific functionality of ERF98 has yet to be investigated. *sl*bHLH59 activates the genes of the pathway in tomato fruits and increases ascorbate levels under the light. The close homolog of this transcription factor (TF) in Arabidopsis is unfertilized embryo sac 12 (UNE12), however, its role for ascorbate biosynthesis has yet to be investigated. Ascorbate undergoes post-translational regulation via constitutive photomorphogenic9-signalosome subunit 5B (CSN5B), VTC3, and the feedback regulation of *VTC2.* CSN5B binds to VTC1 and promotes its degradation under the dark. VTC3 is a putative kinase/phosphatase for light regulation of ascorbate. Feedback regulation of ascorbate is controlled by an unusual open reading frame (uORF), located upstream of the *VTC2* gene. uORF functionality under the light needs further investigations. Ascorbate is also controlled via photosynthetic and mitochondria electron transport chains, designated as photosynthetic electron transport chain (pETC) and mitochondrial electron transport chain (mETC), respectively. This relationship is bidirectional.

Recently, implementing genome wide association study (GWAS) on 302 tomato accessions identified a basic helix–loop–helix (bHLH) transcription factor, *Sl*bHLH59, which positively regulates ascorbate content in tomato fruits [78]. The most similar protein to *Sl*bHLH59 in Arabidopsis appears to be unfertilized embryo sac 12 (UNE12), regulating fertilization [78]. Further investigations are, however, needed to clarify whether this protein has links to the accumulation of ascorbate in Arabidopsis. The schematic representation of the regulatory factors is depicted in Figure 2.

Other components linked to ascorbate regulation have been proposed in multiple studies that are beyond the scope of this review because their roles in acclimation responses have not been fully characterized. Readers are referred to other comprehensive reviews covering the general regulatory components of ascorbate biosynthesis [67,79,80].

### 4.2. Post-Translational Regulation

Further studies support that light regulation of ascorbate occurs post-translationally via the three so far identified mechanisms: (i) through modulating the stability of GDP-man pyrophosphorylase (GMP/VTC1) in light/dark; (ii) through feed-back regulation of GGP (VTC2); and (iii) through a putative kinase::protein phosphatase, VTC3 (Figure 2). Constitutive photomorphogenic9-signalosome subunit 5B (CSN5B) protein has been identified as a component in light/dark regulation of ascorbate [81]. CSN5B interacts with GDP-man pyrophosphorylase (VTC1), and promotes its degradation under dark through the 26S proteasome pathway, resulting in lower ascorbate content. As mentioned above, GGP has been determined as the main regulatory point in the ascorbate biosynthetic pathway, which undergoes rapid feedback control in conditions where ascorbate levels increase [82]. An unusual open reading frame (uORF), starting with an uncommon start codon, ACG instead of AUG, has been found in the 5′-untranslated region (UTR) of GGP, which, under a high concentration of ascorbate, gets translated into a 60–65 residue peptide and further inhibits the translation of *VTC2* [82]. Interestingly, this uORF exists in a variety of plant species [82]. Although it has been hypothesized that this post-translational control might be a causative mechanism in light regulation of ascorbate, further experiments are required to validate this hypothesis. GGP has also been revealed as an important rate-limiting enzyme in *Chlamydomonas*, however, it lacks the feedback regulation mechanism existing in land plants [66].

A deeper investigation of the ascorbate deficient mutant *vtc3* led to the identification of its novel causative loci, encoding VTC3 protein, suggested as a putative kinase/phosphatase for light regulation of ascorbate [83]. Although the protein structure of the VTC3 has been proposed, harboring a kinase and a phosphatase domain at its N-terminal and C-terminal site, respectively, validation of its molecular mechanism has so far remained elusive. Given that the *vtc3* mutants were unable to accumulate ascorbate under continuous light, and localized to the chloroplast, a dual signaling function of the protein in light regulation of ascorbate was suggested by the authors. However, this remains to be experimentally validated [83].

## 5. Role of Sugars, Photosynthesis, and Respiration in Light Regulation of Ascorbate

Given that carbohydrates are substrates for ascorbate synthesis, attempts have been made to decipher the putative links between these metabolites in light acclimation responses. Schmitz et al. [21] examined the roles of sugar and starch metabolism in the acclimation process to high light by using Arabidopsis mutants deficient in either the triose phosphate/phosphate translocator (*tpp*) or ADP-glucose pyrophosphorylase (*AGPase*) or both of them (*adg1-1/tpt-2*). While soluble sugars, mainly glucose, accumulated in both wild type and mutant plants within four hours of high light exposure, the acclimation response was impaired in the mutants after only two days. The comparison of transcriptomic results with publicly available ones revealed a correlation between responses to high light and those to sugar levels following four hours of high light treatment, while the responses at 48 h were also similar to those of ROS accumulation. These results suggest that soluble sugars act as modulators in the short term, but this role is replaced by ROS in the long term. Ascorbate levels increased in all lines over time upon exposure to high light. Interestingly, the redox state of ascorbate was not affected in the triose phosphate/phosphate translocator (TPT) mutant, but reduced in all other lines, suggesting the effect of sugar localization on the redox state of ascorbate [21]. These results emphasize the signaling functions of soluble sugars in high light acclimation and, further, the involvement of ascorbate redox state in signaling pathways.

Although it has been identified that sugars affect the redox state of ascorbate in light acclimation, as described above, regulation of ascorbate accumulation under light appears to be independent of sugars, but dependent on the photosynthetic electron transport chain [59]. Leaf ascorbate levels and transcript levels of the ascorbate biosynthetic genes, *GMP*, *GPP*, *GDH*, and *VTC2*, were decreased, and plants were unable to accumulate ascorbate upon inhibition of photosynthetic electron transport by 3-(3,4-dichlorophenyl)-1,1-dimethylurea (DCMU) and atrazine (ATZ) treatment even under continuous light [59]. In the same study, the effect of sugars on foliar ascorbate levels was examined by transferring two-week-old Arabidopsis seedlings to the media in the presence or absence of sucrose, with subsequent transferal to the dark for 48 h to reduce the internal carbon sources and total ascorbate levels. In effect, darkness led to a significant decline in leaf sugar levels by 90% in both the presence and absence of sucrose, accompanied by a reduction in ascorbate levels. External supplementation of sucrose did not restore the leaf ascorbate pool sizes. In an opposite way, the levels of sugar increased in both the presence and absence of sucrose after transferring them from the dark to the light, and again, the levels of ascorbate could not be restored to normal levels in the sucrose supplemented plants [59]. This observation is in line with the research where the absence of a correlation between carbohydrates and ascorbate levels could be observed in the ripening period of tomato fruits under irradiances that stimulate ascorbate biosynthesis [84]. Despite a remarkable increase in ascorbate levels upon ripening under light, carbohydrate levels remained unchanged. Similarly, alteration in carbohydrate levels upon flower pruning did not show effects on ascorbate levels [84]. Furthermore, these authors confirmed in separate studies that high light has positive effects on ascorbate upregulation only in green tomato fruits, determining photosynthesis as an integral part of this mechanism [84]. Therefore, it appears that photosynthesis is a key component in controlling the leaf ascorbate pool size under light, however, carbon supply through photosynthesis appeared not to be the determinant of the ascorbate levels in Arabidopsis and tomato. Further studies are required to explore the signaling mechanisms in this process. Yet, it has been proposed that the effects of sugars on ascorbate is a genotype-specific phenomenon, which varies in different plant species [73,85]. In contrast to what was observed in the above-mentioned studies, it has been revealed that sucrose feeding in tomato fruits increased the expression of key ascorbate biosynthetic genes such as *VTC1*, *VTC2*, *GDH*, and *GLDH*, as well as of recycling and turnover genes *APX*, *MDHAR*, *DHAR*, and *GR*, pointing to yet unknown signaling components in modulating the ascorbate biosynthetic and recycling gene expression patterns mediated by sugars [86]. That being said, sucrose and glucose feeding had no effects on ascorbate levels in barley and pea embryonic axes [73]. As sucrose feeding in these plant species has not been examined in light shift experiments, it is, however, difficult to draw a solid conclusion on the role of sugars on light regulation of ascorbate.

Given that GLDH, the last enzyme of the ascorbate pathway, lies in the inner membrane of the motochondria [87], being designated as part of the complex I of the respiratory electron transport chain [88], the relationship between ascorbate biosynthesis and respiration is rendered inevitable [73]. Bartoli et al. [87] observed that isolated mitochondria from potato leaves were able to synthesize ascorbate from L-GaL, and subsequently, L-GaL stimulated mitochondrial electron transport rates. Besides, it has been demonstrated that cytochrome C (cytC), located between complexes III and IV, is the electron acceptor of GLDH and treatment of intact mitochondria with potassium cyanide (KCN, an inhibitor of respiration) blocked ascorbate production [69,87]. Following that, Bartoli et al. [69] examined the effects of high light and respiration on ascorbate synthesis in Arabidopsis WTs and transgenic plants overexpressing the mitochondrial alternative oxidase [69]. It has been observed that plants under high light had a higher amount of ascorbate, GLDH, cytochrome C, and cytochrome C oxidase (CCO) activities, accompanied by an improved capacity of the AOX and CCO electron transport rates. Furthermore, *AOX*-overexpressing lines exhibited higher ascorbate levels than WT, especially at high light [69]. AOX is an enzyme in the plant mitochondria that bypasses cytC by directly accepting the electrons from the ubiquinone pool, which prevents over-reduction of the respiratory electron transport chain, and reduces the risk of ROS overproduction [69,73,89]. These studies demonstrate an important mechanism in light regulation of ascorbate through the AOX pathway and further highlight important bidirectional interconnections between the mitochondrial electron transport chain and ascorbate biosynthesis, through both cytC and AOX respiratory pathways.

## 6. Role of Ascorbate in Photosynthesis Coordination of the Energy Systems of the Mitochondria and Chloroplast

Given that ascorbate production is tightly associated with the mitochondrial electron transport chain and considering the well-documented facts on having profound bidirectional relationships with the rates of photosynthesis via a range of mechanisms, we assume that ascorbate takes part in the coordination of the energy systems between the mitochondria and chloroplast.

This hypothesis is raised from validations on ascorbate roles in mitochondrial electron transport rate (mETC) discussed above, photosynthesis, and TCA cycle regulatory networks [39]. Moreover, genes of the Ascorbate-glutathione cycle (ASC–GSH) cycle are expressed in both chloroplast and mitochondria [90], thus both organelles must coordinately take part in ascorbate biosynthesis and recycling. It has been demonstrated in multiple studies that ascorbate can elevate the rate of photosynthesis by a variety of mechanisms, especially in response to acclimation to high light [46,91,92]. These mechanisms include involvement of ascorbate in protecting against photoinhibition in the water–water cycle by scavenging superoxide and hydrogen peroxide, dissipating excess energy, and contribution to thylakoid acidification leading to the control of PSII activity [93,94,95]. Ascorbate is also considered as an alternative electron donor for PSII, whereby it prevents photo-oxidation [96,97]. The ascorbate-redox state is also known to affect photosynthetic activity through guard cell signaling and stomatal movement and also through changing the expression of the nuclear and chloroplastic encoded genes [40]. Furthermore, feeding Arabidopsis *vtc1* mutants with ascorbate had a great impact on photosynthetic gene expression, leading to an increased and decreased expression of some of the chloroplast- and nuclear-encoded genes, respectively [98].

However, recently observed differences in the photosynthetic responses of *vtc2-1* and *vtc2-4* mutants under high light raised the hypothesis that higher susceptibility of the *vtc2-1* mutants to photoinhibition, previously reported in the literature [46,92], might not have been caused by the lower ascorbate levels in the mutants [99]. Unlike *vtc2-1,* which carries a point mutation, the *vtc2-4* mutant is a T-DNA insertion line with a complete loss of function [99]. Contrary to *vtc2-1*, the *vtc2-4* mutants have unchanged levels of zeaxanthin contents and, despite having similar levels of NPQ under high light (lower than the WT), *vtc2-1* had greater photochemical quenching in the dark (qP_d_) values than the WT. Therefore, the authors suggested ascorbate as an essential component for growth, but not for photoprotection [99].

Intriguingly, observations on transgenic tomato plants antisensed in mitochondrial malate dehydrogenase (*mdh*), strengthening the hypothesis of ascorbate acting in the coordination of the energy systems of the mitochondria and chloroplast [13,100]. The reduction of the TCA cycle occurring in these transgenic lines via down-regulation of the expression of the mitochondrial MDH did not affect the respiration, but resulted in a fourfold increase in ascorbate following an upregulation of the activity of GLDH. Detailed studies revealed that this was because flux, through the GLDH activity, was upregulated in these lines, with the consequence that electrons were supplied to the mitochondrial electron transport chain [100]. Furthermore, incubating tomato leaf discs with ascorbate under constant illumination increased the amount of carbon assimilation and starch levels [100]. This further strengthens the link between chloroplast and mitochondria in ascorbate levels. Evaluation of tomato plants in which the GLDH was reduced in expression by RNA interference had dramatic consequences on both plant and fruit growth and development [101]. This observation is, however, complicated by a report of Tomaz et al. [102], in which they examined the *mdh* double mutants in Arabidopsis. The Arabidopsis *mdh* double mutants had increased levels of ascorbate, but higher levels of mitochondrial respiration and a lower activity of GLDH, contrary to what had been observed in tomato plants [102]. More investigations are needed to clarify the cause of this difference observed between the two species.

In conclusion, considering all the described effects of ascorbate on photosynthesis and vice versa, and its intimacy to the respiratory electron transport chain, ascorbate can be considered as an important component in plant central metabolism, modulating the energy systems between the chloroplast and mitochondria [39]. However, the molecular mechanisms and the signals responsible for these network of interactions need further investigations.

## 7. Thiamin Biosynthesis

**Figure 3:**
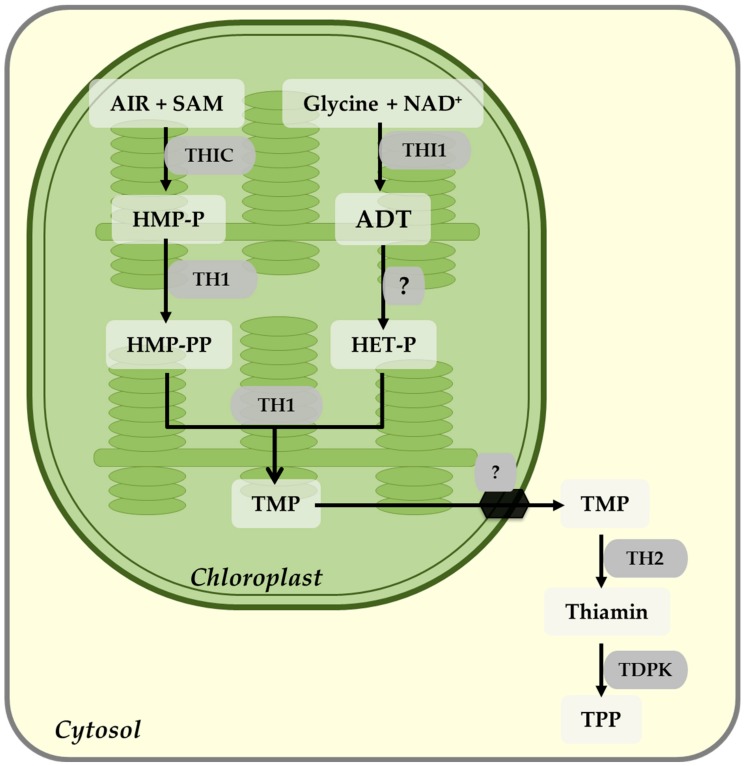
Thiamin biosynthesis pathway in plants. Thiamin is synthesized from pyrimidine and a thiazole moiety, both of which are synthesized in the chloroplast. The detailed description of the pathway is presented in the text. Abbreviations of the substrates: 5-aminoimidazole ribonucleotide (AIR), S-adenosylmethionine (SAM), nicotinamide adenine dinucleotide (NAD^+^), 4-amino-2-methyl-5-hydroxymethylpyrimidine phosphate (HMP-P), adenylated thiazole intermediate (ADT), 4-methyl-5-b-hydroxyethylthiazole phosphate (HET-P), thiamin monophosphate (TMP), thiamin pyrophosphate (TPP). Abbreviations of the enzymes: thiamin C synthase (THIC), -methyl-5-b-hydroxyethylthiazole phosphate (HET-P) synthase (THI1), thiamin monophosphate pyrophosphorylase (TH1), haloacid dehalogenase (HAD) family phosphatase (TH2), TPP kinases (TDPK).

In plants, thiamin is synthesized from pyrimidine and a thiazole moiety, both of which are synthesized in the chloroplast. Synthesis of pyrimidine moiety is catalized by thiamin C synthase (THIC) by converting 5-aminoimidazole ribonucleotide (AIR) and S-adenosylmethionine (SAM) as substrates to 4-amino-2-methyl-5-hydroxymethylpyrimidine phosphate (HMP-P) [103,104,105]. Thiazole moiety is synthesized by the action of 4-methyl-5-b-hydroxyethylthiazole phosphate (HET-P) synthase (THI1) [106], catalizing the conversion of nicotinamide adenine dinucleotide (NAD+) and glycine as substrates to an adenylated thiazole intermediate (ADT) [107,108,109]. ADT is then hydrolyzed to HET-P, by an uncharacterized enzyme. Phosphorylation of HMP-P to HMP-PP, and subsequently condensation of HMP-PP and HET-P, is done by the action of a bifunctional enzyme, thiamin monophosphate pyrophosphorylase (TH1), which eventually leads to the formation of thiamin monophosphate (TMP) [30,110]. TMP is dephosphorylated to thiamin by a haloacid dehalogenase (HAD) family phosphatase (TH2) [111], and subsequently pyrophosphorylated to thiamin pyrophosphate (TPP) by TPP kinases (TDPKs), which are located in the cytosol (Figure 3) [110,112].

In the past years, different attempts have been made to biofortify staple crops with thiamin [112,113,114]. These efforts were possible thanks to a recent increase in the understanding of thiamin metabolism in plants. Several genes involved in thiamin biosynthesis, regulation, transport, and salvage have been identified owing to the availability of complete plant genome sequences [30,115,116]. Although this determined the beginning of thiamin engineering strategies, several aspects of thiamin metabolism are still unclear [114].

### Regulation of Thiamin Biosynthesis

THIC promoter has been identified to be essential for the circadian clock-regulation of thiamin biosynthesis [25]. Thiamin levels are also regulated by a TPP responsive riboswitch located at the 3’ region of the THIC pre-mRNA, controlling the stability of THIC mRNA [25]. Riboswitches are mRNA sensors that bind small molecules and consecutively regulate gene expression. The TPP riboswitch mediates a feedback regulation of the thiamin biosynthesis pathway. When TPP levels are high, TPP binds to the riboswitch leading to intron splicing and instable THIC mRNA, which is consequently degraded [24,117].

Another layer of regulation relies on the activities of thiamin biosynthesis enzymes. THI1 is a single turnover protein; it loses functionality once the cysteine residue is used in the reaction [107,118], and thus a single step of thiamin biosynthesis requires high energy levels. In addition, excess HMP-PP could work as an inhibitor for TH1 activity, as suggested by *THIC*-overexpressing plants [119]. Bioengineering studies for the fortification of crops also showed that an increase in expression of *THIC* and *THI1* in Arabidopsis and rice plants does not necessarily result in an increase in thiamin production [112,114,120]; these enzymes rely on factors, such as reduction by thioredoxins for THIC and THI1, and supply of sulfur for THIC’s iron-sulfur cluster [104,114,121], which could also be limiting.

**Figure 4:**
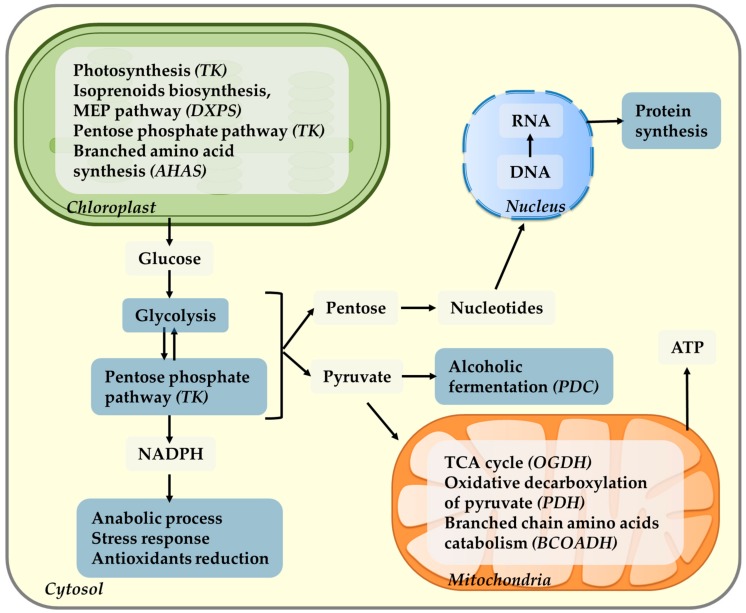
Cell localization of the main thiamin dependent-enzymes and its participation in metabolic pathways. Thiamin pyrophosphate (TPP), the active form of thiamin, works as an essential coenzyme for the enzymes involved in photosynthesis in chloroplasts, pentose phosphate pathway, and alcoholic fermentation in cytoplasm, as well as in ATP synthesis in the participation in oxidative decarboxylation of pyruvate and tricarboxylic acid cycle in mitochondrial central metabolism. Thiamin has also been shown to be involved in the acclimation responses to abiotic stresses; photoperiod; and working directly as an antioxidant, scavenging ROS; a protection molecule; and indirectly by contributing to the cell energy poll, conferring the cell the necessary metabolic flexibility to acclimate to new conditions. The thiamin-dependent enzymes shown are α-ketose transketolase (TK); 1-deoxy-D-xylulose-5-phosphate synthase (DXPS); acetohydroxyacid synthase (AHAS); pyruvate dehydrogenase (PDH); 2-oxoglutarate dehydrogenase (OGDH); and branched chain 2-oxoacid dehydrogenase (BCOADH). MEP, methylerythritol pathway.

The complexity of thiamin biosynthesis regulation and the universality of thiamin-requiring enzymes across kingdoms and their association with core metabolic pathways suggest a crucial role of TPP in the regulation of cellular metabolism and the potentially prejudicial effect of inappropriate total thiamin levels. The cell localization of the main thiamine-dependent enzymes and its participation in metabolic pathways are depicted in Figure 4.

## 8. Thiamin and Chloroplast Interactions

In the chloroplast, TPP-dependent enzymes play a role in photosynthesis (α-ketose transketolase, TK, and 1-deoxy-D-xylulose-5-phosphate synthase, DXPS), pentose phosphate pathway (TK), and branched amino acid synthesis (acetohydroxyacid synthase, AHAS). The primary pathway for carbon fixation in plants is the Calvin–Benson cycle (also known as the C_3_ cycle), which is connected to several other pathways via its intermediates. TK has a central location in the Calvin–Benson cycle catalyzing the reversible transfer of a molecule with two carbons from sedoheptulose 7-phosphate to glyceraldehyde 3-phosphate (G3P), generating xylulose 5-phosphate (Xu5P) and ribose 5-phosphate, or from fructose 6-phosphate to produce Xu5P and erythrose 4-phosphate. These reactions are essential for the regeneration of ribulose 1,5-bisphosphate in the Calvin–Benson cycle maintaining active photosynthetic rates and providing precursor molecules for the shikimic acid pathway and phenylpropanoid metabolism (erythrose 4-phosphate) [122].

Additionally, TK is the main TPP-dependent enzyme in the pentose phosphate pathway. Different from the Calvin–Benson cycle, which utilizes CO_2_, NADPH, and ATP to produce hexose sugars, the pentose phosphate pathway utilizes hexose substrates to produce NADPH and pentoses while releasing CO_2_. Through participation in the pentose phosphate pathway, transketolase has three important functions in the metabolism of the cells: (i) provision of pentoses for the synthesis of nucleotides; (ii) provision of metabolites for glycolysis or gluconeogenesis pathways; and (iii) indirectly influencing the synthesis of NADPH, required for the anabolic processes and antioxidants reduction (glutathione, ascorbate). Consequently, an appropriate activity of transketolase is essential for the proper functioning of lipid and carbohydrate metabolism [27].

Chlorophyll and carotenoids are synthesized in the methylerythritol pathway (MEP) and the first reaction of this pathway is catalyzed by DXPS; this enzyme combines G3P from the Calvin–Benson cycle with pyruvate from the glycolytic pathway to form deoxyxylulose 5-phosphate (DXP) [26]. Additionally, DXPS is a TPP-dependent enzyme and the product of the DXPS reaction, DXP, is the first substrate in the biosynthesis of the hydroxyethylthiazole phosphate (HETP) moiety of the thiamin molecule itself, and thus TPP [123].

The acetohydroxyacid synthase (AHAS, also known as acetolactate synthase, ALS) is also a TPP-dependent enzyme. It catalyzes the first reaction in the synthesis of the branched-chain amino acids, valine, leucine, and isoleucine, which are only produced in plants [124]. This enzyme is responsible for converting two molecules of pyruvate into 2-acetolactate, the first reaction in a three-step pathway used to produce the three amino acids [124].

## 9. Thiamin and Mitochondria Interactions

Thiamin participates in the mitochondria central metabolism by functioning as a cofactor forpyruvate dehydrogenase (PDH), 2-oxoglutarate dehydrogenase (OGDH), and branched chain 2-oxoacid dehydrogenase (BCOADH, or branched chain ketoacid dehydrogenase, BCKDH). The PDH complex has a central role in bioenergetic processes, controlling the supply of acetyl-CoA into the TCA cycle and anabolic reactions, linking glycolysis and the TCA cycle via the oxidative decarboxylation of pyruvate [27,125]. PDH also produces acetyl-CoA from pyruvate in the chloroplast, which is used in the synthesis of fatty acids [27].

In the TCA cycle itself, OGDH catalyzes a rate-limiting step [27,125]. It converts 2-oxoglutarate, coenzyme A, and NAD^+^ to succinic acid, while releasing NADH and CO_2_ as part of the process. It has also been shown in several studies to be a key regulation point in plant metabolism [126,127,128]. Additionally, it is responsible for the distribution of succinyl-CoA and 2-oxoglutarate for substrate level phosphorylation of GDP, ADP, or for the synthesis of several amino acids and heme group [27,129].

Another TPP-dependent enzyme, BCOADH, catalyzes the breakdown of the branched chain amino acids, and studies have shown its important role in amino acid metabolism in Arabidopsis [130]. In addition, the enzyme pyruvate decarboxylase (PDC) in the cytosol also requires thiamin as a cofactor. It functions to break down pyruvate to acetaldehyde, an essential step for energy production via alcoholic fermentation under anoxia in Arabidopsis [131,132].

## 10. The Role of Thiamin in Mediating Plant Fitness and Acclimation

In addition to its role in central metabolism, studies in different plant species have shown that alterations in the levels of thiamin vitamers result in smaller plants, chlorosis of the leaves, growth retardation, delayed flowering, fitness cost, and an influence on yield penalty [25,26,105,111,133,134]. Some of these phenotypes, such as chlorosis and the delayed flowering, have also been shown to be dependent on the light regime [25,32].

The functions of thiamin in the regulation of the metabolic networks during photoperiod transition were deeply investigated in our group [32]. While control plants display changes in the amplitude of diurnal oscillation in the levels of metabolites, TPP riboswitch mutant plants with high levels of TPP do not show such metabolic flexibility. The results also indicate a close relationship between photorespiration and the TCA cycle as the mutant plants accumulate less photorespiratory intermediates such as glycine, serine, and glycerate [32].

Thiamin biosynthesis has also been well documented to be activated when plants are exposed to abiotic stresses [28,31,135]. Rapala-Kozik and co-authors (2008; 2012) demonstrated that thiamin biosynthesis is activated on the acclimation response of Arabidopsis to salt, osmotic, and oxidative stress [29,30]. These stresses induce the expression of genes for TPP biosynthesis and thiamin dependent-enzymes, resulting in increased levels of thiamin and TPP, those which can consequently be incorporated into the requiring enzymes associated with central metabolic pathways, as described previously. Continuous abiotic stress such as high salinity and sugar deprivation was also shown to increase thiamin biosynthesis gene expression [30,136]. Interestingly, flooding/hypoxic conditions also impact thiamin biosynthesis expression patterns in roots. Under low O_2_ supply, roots switch from respiration to pyruvate fermentation, and high levels of thiamin could be required in this alternative route, as TPP acts also as a cofactor for PDC present in the fermentative metabolism [136].

Proteomics and transcriptomics studies in other species have also detected significant changes in thiamin biosynthesis and thiamin dependent enzymes during heat, drought, and cold stress conditions [137,138]. Changes in the protein levels associated with thiamin are transient, with increased abundance at early stages of stress followed by a decrease in protein levels associated with thiazole synthase in the thiamin biosynthesis pathway [137]. These conclusions are in agreement with our results describing the importance of thiamin levels for metabolic flexibility during acclimation [32]. Thiamin is also known as a potent antioxidant and a crop protection molecule in plants, playing important roles in plant acclimation [17]. It has been shown that thiamin has the antioxidant capacity by O^2−^/OH^−^ scavenging and also recycling of vitamin C through the synthesis of NADPH [17,33]. Further, it has been revealed that paraquat-treated Arabidopsis, supplied by thiamin had reduced oxidative stress compounds, protein carbonyls, and dichlorofluorescein. It has also been shown in this study that Arabidopsis plants accumulated higher levels of TMP and TPP after exposure to high light, low temperatures, and osmotic and salt stress [31]. Yet, further investigations of thiamin in plants are needed to clarify whether it functions as an antioxidant directly or indirectly by supplying NADH and NADPH [17].

Although more scarce, studies on the function of thiamin during biotic stress have shown that treating different species with thiamin activates the systemic acquired resistance (SAR) and resistance to pathogen attack, such as fungal, bacterial, and viral infections. The thiamin treatment activates pathogen-related genes (PR) and stress signalling hormones, abscisic acid (ABA), and jasmonates, enhancing pathogen resistance in plants [135,139].

Taken together, these studies implicate that thiamin and its vitamers, despite being present at a very low concentration, play a general role in central metabolism and in acclimation responses. Indeed, their low concentrations are even representative of known signaling compounds. These results also support the idea that signaling molecules not only coordinate the expression of nuclear and organelle genes, but also maintain cellular functions at optimal levels in response to changes in environmental conditions [15,140,141].

## 11. Concluding Remarks

In this review, we addressed the current knowledge on the roles of ascorbate and thiamin in plant metabolism with the emphasis on plant acclimation responses, specifically to high light and photoperiod acclimation, regarding ascorbate and thiamin, respectively.

In brief, ascorbate has important roles in modulating the energy systems between the chloroplast and mitochondria during high light acclimation. Incorporation of GLDH, the ultimate enzyme of the pathway, into the mitochondrial electron transport chain is a rationale for considering the tight association of ascorbate biosynthesis and mitochondria metabolism. This relationship is found to be bidirectional as mETC has a positive regulatory role on ascorbate biosynthesis through both AOX and cytC respiratory pathways. The existence of such a relationship might guarantee the balance of the electron flow under environmental stresses [39]. Likewise, photosynthesis regulates ascorbate pool size under the light. However, carbohydrates are direct substrates for ascorbate biosynthesis; their role in light regulation of ascorbate remains ambiguous and appears to be species-specific. The relationship between ascorbate, respiration, and photosynthesis is bidirectional. In effect, ascorbate elevates the rate of photosynthesis by a variety of mechanisms in response to acclimation to high light, such as through the water–water cycle, by scavenging ROS, dissipating excess energy through the xanthophyll cycle, donating electrons to PSII, guard cell signaling, and stomatal movement. It also regulates the nuclear and chloroplastic encoded genes of the photosynthesis.

**Figure 5:**
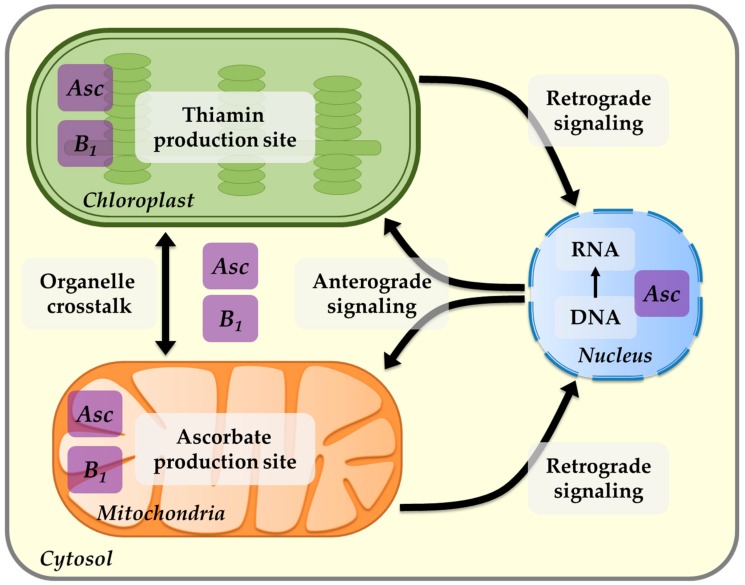
Schematic illustration of plant intracellular communication. Anterograde (nucleus to organelle) and retrograde (organelle to nucleus) signaling pathways, as well as the main active site of ascorbate (Asc) and thiamin (B_1_) as signaling molecules, are shown. The ubiquitous existence of ascorbate and thiamin in cellular organelles, as well as the tight interconnection of the two vitamins between chloroplast and mitochondria, points to their important roles in the crosstalk between the two organelles.

Furthermore, in this review, we addressed the complexity of thiamin biosynthesis regulation, the universality of thiamin-requiring enzymes across kingdoms, and their association with core metabolic pathways. Therefore, a general role of TPP in the regulation of cellular metabolism and the acclimation process can be considered. A schematic description of the organellar communication and the involvement of ascorbate and thiamin is depicted in Figure 5.

Despite the thus far identified physiological dependencies of the two vitamins on the key players of metabolism, photosynthesis, and respiration and their roles in optimizing their activities, the underlying signaling and genetic factors in this process have remained a challenge for future research. QTL mapping and GWAS can be considered as alternatives to fill this gap.
